# Frontal GABA Levels Change during Working Memory

**DOI:** 10.1371/journal.pone.0031933

**Published:** 2012-04-02

**Authors:** Lars Michels, Ernst Martin, Peter Klaver, Richard Edden, Fernando Zelaya, David J. Lythgoe, Rafael Lüchinger, Daniel Brandeis, Ruth L. O’Gorman

**Affiliations:** 1 Center for MR-Research, University Children’s Hospital, Zurich, Switzerland; 2 Institute of Neuroradiology, University Hospital Zurich, Zurich, Switzerland; 3 Zurich Center for Integrative Human Physiology, Zurich, Switzerland; 4 Division of Abnormal Psychology and Clinical Intervention, Institute of Psychology, University of Zurich, Zurich, Switzerland; 5 Russell H. Morgan Department of Radiology and Radiological Science, The Johns Hopkins University School of Medicine, Baltimore, Maryland, United States of America; 6 F.M. Kirby Center for Functional Brain Imaging, Kennedy Krieger Institute, Baltimore, Maryland, United States of America; 7 Centre for Neuroimaging Sciences, Institute of Psychiatry, King’s College London, London, United Kingdom; 8 Department of Child and Adolescent Psychiatry, University of Zürich, Zurich, Switzerland; 9 Department of Child and Adolescent Psychiatry and Psychotherapy, Central Institute of Mental Health, Medical Faculty Mannheim, Heidelberg University, Mannheim, Germany; University of Bern, Switzerland

## Abstract

Functional neuroimaging metrics are thought to reflect changes in neurotransmitter flux, but changes in neurotransmitter levels have not been demonstrated in humans during a cognitive task, and the relationship between neurotransmitter dynamics and hemodynamic activity during cognition has not yet been established. We evaluate the concentration of the major inhibitory (GABA) and excitatory (glutamate + glutamine: Glx) neurotransmitters and the cerebral perfusion at rest and during a prolonged delayed match-to-sample working memory task. Resting GABA levels in the dorsolateral prefrontal cortex correlated positively with the resting perfusion and inversely with the change in perfusion during the task. Further, only GABA increased significantly during the first working memory run and then decreased continuously across subsequent task runs. The decrease of GABA over time was paralleled by a trend towards decreased reaction times and higher task accuracy. These results demonstrate a link between neurotransmitter dynamics and hemodynamic activity during working memory, indicating that functional neuroimaging metrics depend on the balance of excitation and inhibition required for cognitive processing.

## Introduction

The average adult brain utilises approximately 20% of the total energy consumption of the body. The manner in which this energy consumption is apportioned among cell types within the brain and the various activities they perform in different regions is a subject of ongoing research [1], [2]. While the majority of cortical neurons are excitatory glutamatergic cells, the remaining 20% of the neuronal population are inhibitory GABA-ergic (gamma-aminobutyric acid) interneurons [3], [4]. An imbalance of excitatory or inhibitory neurotransmitter levels is thought to underlie several developmental and clinical disorders such as attention deficit hyperactivity disorder (ADHD), Parkinson’s disease, schizophrenia and epilepsy [5], [6], [7], [8]. Neurotransmitter activity is also thought to be related to the hemodynamic changes associated with brain activation, but the precise relationship between neurotransmitter levels and cerebral blood flow (CBF) has not yet been established.

Both GABA and Glx (a combined measure of glutamate (Glu) and glutamine (Gln)) can be reliably detected in the human brain with magnetic resonance spectroscopy (MRS) [9], [10], and recent studies have uncovered a link between resting GABA levels and the blood oxygen level dependent (BOLD) functional magnetic resonance imaging (fMRI) signal [11], [12], [13], the resting perfusion, and changes in cerebral blood volume during visual stimulation [13]. Changes in GABA have also been observed with motor learning [14] and during transcranial magnetic stimulation [15], [16], and changes in glutamate have been detected following acute pain stimulation [17], [18] and during visual stimulation [19], suggesting that MRS measures of GABA and glutamate are sensitive not only to baseline neurotransmitter levels but also to regional modulations of task-related neurotransmitter activity.

MRS experiments have also shown that most of the energy expended during functional activity is generated by glucose oxidation [20], [21]. The rate of glial glucose uptake appears to be closely coupled to the glutamate/glutamine cycle, demonstrating a stoichiometry of approximately 1∶1 across a range of levels of cortical electrical activity [22], [23], [24]. While the coupling between perfusion and metabolism is disrupted during major changes in brain activity (e.g., the early phase of stimulation) [25], perfusion is thought to be at least partially regulated by neurotransmitter-mediated signalling [26], [27]. Functional imaging metrics like the BOLD signal or changes in perfusion may therefore represent indirect markers for changes in neurotransmitter flux. However, to date regional modulations of GABA and Glx levels have not been demonstrated in vivo during a cognitive task, and the relationship between changes in neurotransmitter and hemodynamic activity during cognition is unknown.

The purpose of this study was to examine in humans whether regional modulations in neurotransmitter concentrations can be detected during cognition with MRS and whether these changes can be related either to the baseline CBF or the increase in CBF during the task. We hypothesized that the resting GABA concentration would be positively correlated to the resting perfusion and inversely correlated to the change in perfusion during the task and that GABA and Glx concentrations would alter significantly during cognition. Further, we examined whether the neurotransmitter concentration was linked to performance (reaction time and task accuracy). Since experimental and modelling data [28], [29], [30], [31], [32], [33], [34], [35], [36] have demonstrated a link between neurotransmitters and both working memory (WM) retention and long-term memory formation we chose to investigate changes in cerebral neurotransmitter levels during a delayed match-to-sample WM task. We selected the left dorsolateral prefrontal cortex (DLPFC) as the target region for spectroscopic measurements, because electrophysiological [37], [38], [39], [40], [41], [42], modelling [37], and brain imaging studies using fMRI [43], [44], [45] and position emission tomography [41], [46], [47] have shown that the DLPFC is engaged in WM processing.

## Methods

### Subjects

The subject group consisted of sixteen healthy right-handed volunteers (7 female, mean age: 28 years, range 25–38) with no history of neurological or psychiatric illness, illegal substance abuse or use of psychotropic medication. All subjects refrained from caffeine, alcohol and nicotine for at least 8 h before the experiment. All subjects gave written informed consent prior to participation. The study falls under the ethical approval (StV 08/08 and 2010–0393/2) of the ‘Kantonale Ethikkommission Zürich’ (http://www.kek.zh.ch).

### MR-data Acquisition

MR imaging and spectroscopy studies were performed with a 3T GE HDxt MRI scanner (GE Medical Systems, Milwaukee, WI, USA) equipped with Twin Speed gradients. The scanning protocol included a 3D fast inversion-recovery prepared gradient echo acquisition (number of slices = 172, slice thickness = 1.2 mm, repetition time (TR) = 10 ms, echo time (TE) = 2.92 ms, field of view = 240 mm×240 mm, flip angle = 20°, matrix = 256×192, voxel resolution: 0.8×0.8×1.2 mm), used for localization of the spectroscopy voxels. The volumetric T1-weighted images were also segmented into grey matter, white matter and CSF maps using statistical parametric mapping (SPM5, Wellcome Dept. of Cognitive Neurology) to correct the spectroscopy results for partial volume CSF contamination.

Resting cerebral perfusion images were acquired with a single shot QUIPSS II arterial spin labelling (ASL) sequence [48], with a PICORE (proximal inversion with control for off-resonance effects) tagging scheme and a gradient-echo EPI readout. The ASL perfusion images were acquired during an eyes closed (EC) rest condition with TR = 3 s, TE = 30 ms, TI_1_ = 600 ms, TI_2_ = 1300 ms. 10 near-axial slices were prescribed with a slice thickness of 5 mm, a slice gap of 1 mm, a field of view of 24 cm, and a matrix of 64×64. In total 60 pairs of control/label image volumes were acquired over a total scan time of 6 minutes.

Following acquisition of the resting ASL perfusion images, five consecutive single voxel edited ^1^H MR spectra were acquired from a 25×40×30 mm^3^ voxel of interest positioned in the left DLPFC using the MEGA-PRESS method [12], [49], [50]. For each spectrum, 320 spectral averages were acquired with a repetition time (TR) of 1800 ms, an echo time of 68 ms, and an eight step phase cycle, resulting in an acquisition time of approximately 10 minutes. MEGA-editing was achieved with 16 ms Gaussian editing pulses applied at 1.9 ppm and 7.5 ppm in alternate spectral lines. The water suppression and shimming were optimised using a standard automated pre-scan, incorporating a first-order automatic shimming method based on a fast 3-planar acquisition of B0 maps, which are used to calculate the appropriate shim values. The average water line width was 8.6 Hz (range 7–10 Hz). The first MEGA-PRESS spectrum was acquired during an EC rest condition, and the following four spectra were acquired during continuous performance of a visuo-verbal Sternberg WM task. During the task, five or seven letters were presented for a stimulus period of 2 seconds, and had to be maintained in memory for a retention period of 5 seconds before a single letter appeared for a probe interval of 2 seconds (task duration: 10 min). Each run consisted of 48 trials with 24 trials for each load condition. Each load condition was shown in six mini-blocks comprised of 4 trials. Subjects had to indicate by button press whether or not the letter was part of the stimulus set. For the purpose of this study load levels of 5 and 7 were selected to make the task challenging [51], [52], [53], but not so difficult that the participants would be unable to perform accurately and continuously for 10 minutes.

**Figure 1:**
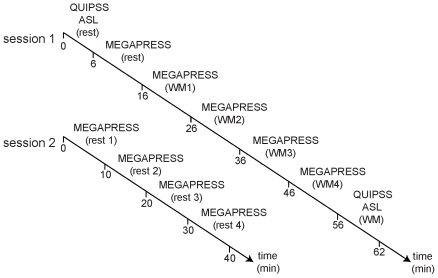
Schematic timeline of the experimental design. ASL: arterial spin labelling, WM: working memory.

In order to achieve a consistent MRS voxel position between subjects and measurement sessions, the voxel was positioned on an imaging slice 1.5 mm above the superior margin of the lateral ventricles. The length of the midline was measured on this slice and the centre of the voxel was defined at a point 1/3 of the distance down the midline from the anterior margin of the brain, and in the centre of the left hemisphere (1/2 of the distance between the midline and the left lateral border of the brain, on a line perpendicular to the midline). For each metabolite spectrum, 16 water reference lines were also acquired as part of the standard GE PROBE acquisition. After collection of the four WM spectra, a second QUIPSS II perfusion image dataset was acquired during continuous performance of the same WM task, with the identical scan parameters to the resting QUIPSS ASL acquisition (*session 1*, Fig. 1).

In order to investigate the baseline stability of GABA and Glx levels over time and to characterise any temporal changes in neurotransmitter levels which might arise independently from the task, four consecutive resting MEGA-PRESS spectra (10 min/run) were acquired from the same anatomically-defined voxel location in the DLPFC in a separate scanning session (*session 2*, Fig. 1). The resting MEGA-PRESS spectra were acquired with alternating eyes open (EO) and EC conditions, with the order (i.e. EC, EO, EC, EO or EO, EC, EO, EC) counterbalanced between participants.

### MR-data Analysis

Coil-combination of the phased array spectra was performed with weighting factors derived from the first point of the unsuppressed water free induction decay signal from each coil. Both GABA and Glx were estimated from the edited spectra, and all MRS data were processed in the frequency domain using LCModel version 6.1-4F [54], using a simulated basis set including basis set spectra for GABA, glutamate, glutamine, Glx, N-acetyl aspartate (NAA) and glutathione. The Cramer-Rao lower bound cut-off (an index of data fitting quality) was set to 10%. The in vivo water-scaled concentrations reported by LCModel were divided by the fractional content of brain tissue (i.e., the percentage[grey matter] + percentage[white matter] in the voxel) to correct for partial volume CSF contamination.

**Figure 2:**
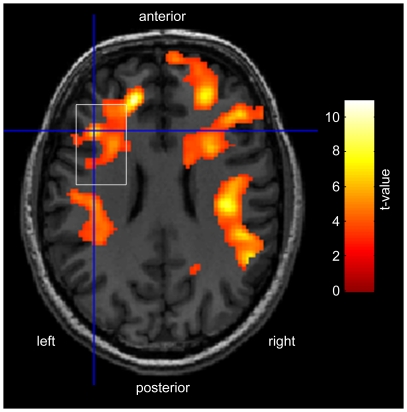
Areas of significant perfusion change during the WM task (p<0.001, uncorrected, k = 150). The location of the left DLPFC voxel (white rectangle) is shown for comparison. Results are presented on an axial slice (MNI z-coordinate = 24) of a T1-weighted image from a single subject.

Perfusion maps were computed using in house software, according to the standard single-compartment QUIPSSII model [48]. The equilibrium magnetisation (M0) of blood was estimated from a region of interest in white matter assuming a ratio of the proton density of blood to that in white matter of 1.06. [48]. The (left) DLPFC perfusion was then calculated within a region of interest defined by the margins of the spectroscopy voxel. In order to estimate the regional specificity of the perfusion changes during WM relative to the position of the MRS voxel, the perfusion maps were normalised in SPM5 and differences in perfusion between the task and rest conditions were assessed with a paired t-test, using a statistical threshold of p<0.001 (uncorrected) with k = 150 voxels. Significant areas of perfusion change across the group are show in figure 2, superimposed on the T1-weighted image from a single subject.

Since we had no a priori hypothesis about the direction of the predicted change in GABA and Glx between the baseline resting state and subsequent task runs, differences in GABA and Glx between the resting and WM spectra were tested using two-tailed t-tests. The specificity of temporal changes in neurotransmitter levels during the repeated task (WM1-WM4, session 1) and rest (R1-R4, session 2) conditions was examined by a repeated-measures analysis of variance (rm-ANOVA). In addition, the fractional change in GABA and Glx for each WM spectrum relative to the baseline resting levels acquired at the start of session 1, and the fractional change in GABA and Glx levels for each resting spectrum relative to the baseline resting levels acquired at the start of session 2 were calculated. In order to investigate the link between perfusion and neurotransmitter levels, correlations between GABA, Glx, the resting perfusion, and the change in perfusion, GABA, and Glx during the task were evaluated using Pearson’s correlation coefficient. Linear regression analyses were performed to examine potential interactions between performance and neurotransmitter concentration. Estimation of the skewness and the kurtosis of the data revealed that the MRS and perfusion measures were normally distributed (skewness and kurtosis values <1.5 and >−1.5).

## Results

### Behavioural Results

The mean (i.e., load independent) task accuracy across all WM repetitions was 78.3±10. 4 % and the mean reaction time (RT) was 927.3±325.5 ms. There was no significant difference in RT between WM1 and WM4 (p = 0.06, paired t-test), but subjects responded faster across WM runs as shown by a main effect of time (F_1,15_ = 4.84, p = 0.05, one-way rm-ANOVA). Further, a linear regression analysis revealed a trend for towards a positive correlation between the mean RT and the GABA (not Glx) concentration (t_15_ = 1.68, p = 0.1). While mean task accuracy was not significantly different between WM1 and WM4 (p = 0.34) or across WM runs (F_1,15_ = 0.78, p>0.05, one-way rm-ANOVA), a linear regression analysis revealed a trend towards an inverse correlation between mean task accuracy and GABA (not Glx) concentration (t_15_ = −1.72, p = 0.092). There were no significant between-load differences for RT and task accuracy (i.e., no main effect of time and load), as tested by a two-way rm-ANOVA with the factors time (four levels) and load (two levels: load 5 and load 7). In addition, no time×load interaction was found for RT and task accuracy (all F’s<1).

### MR Results

**Figure 3:**
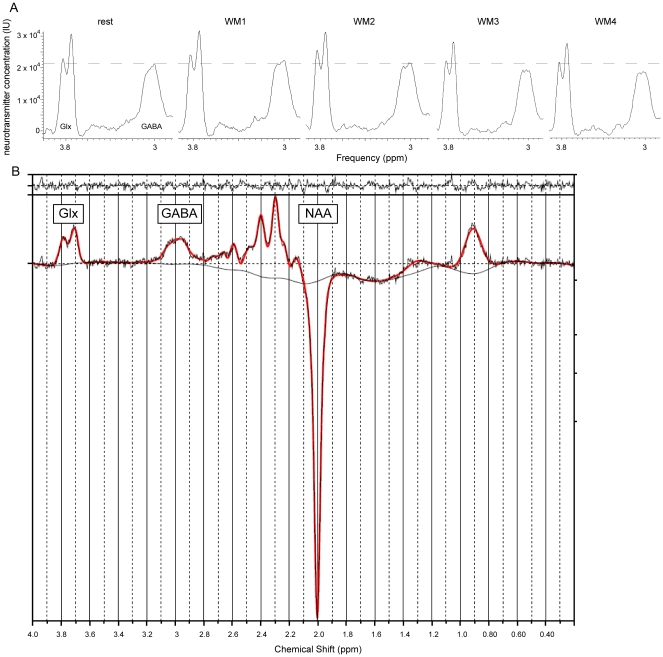
Representative MEGA-PRESS spectra. (A) Averaged MEGA-PRESS spectra (averaged across the subject group) acquired at rest (left) and during the WM task. The GABA peak at 3.0 ppm appears to increase between the resting spectrum and the first WM spectrum, and then decrease during performance of the WM task (panels 2-5). The dashed line marks the resting state peak. Glx: glutamate + glutamine concentration, GABA: gamma-aminobutyric acid, NAA: N-acetylaspartate, IU: institutional units. (B) LCModel output for a single subject: the fit is shown in red, superimposed on the edited spectrum (in black). The top panel shows the residuals between the MRS data and the spectral fit.

Representative GABA-edited spectra from the group and from a single subject are shown in Fig. 3A and Fig. 3B, respectively, and significant differences between the WM task and rest-related perfusion are shown in figure 3.

#### Perfusion

Significant increases in perfusion are observed in the DLPFC (BA 9), middle frontal gyrus (BA 6), insular cortex (BA 13), and the pre- and postcentral gyri during WM relative to rest.

#### GABA

**Figure 4:**
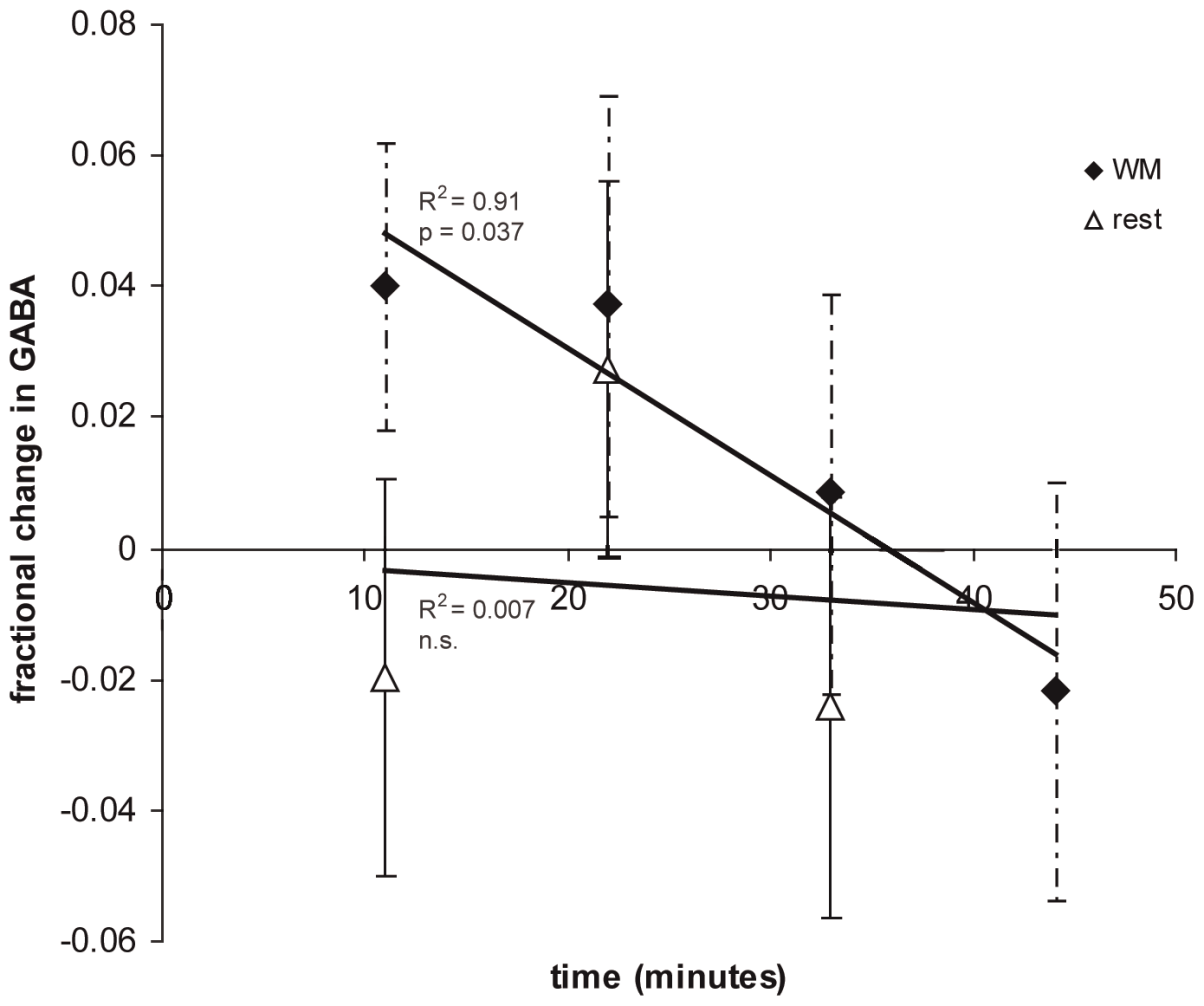
Fractional change in GABA during the WM task, relative to the baseline resting level at time = 0. The fractional change in GABA during the consecutive resting spectra is shown for comparison.

For session 1, the average GABA levels across the subject group showed a significant initial increase during WM1 relative to the baseline resting level (t_15_ = 2.2, p = 0.044), followed by a significant decrease in concentration across the subsequent WM runs (one-way rm-ANOVA: main effect of time: F_1,15_ = 5.14, p = 0.037). The decrease in GABA across the four WM task runs showed a strong negative linear correlation (R^2^ = 0.91, Fig. 4). No effect of time (one-way rm-ANOVA: F_1,15_ = 0.26, p = 0.62) and no correlation was observed between the repeated resting measurements of session 2 (R^2^ = 0.007, Fig. 4). Further, to evaluate whether a decrease in GABA is specific for WM we used a rm-ANOVA with the factors session (WM and rest) and time (4 time points, where the first rest in session 1 was excluded from the analysis). This test revealed a main effect of session (F_1,15_ = 5.23, p = 0.038), a trend to significance for a main effect of time (F_1,15_ = 3.4, p = 0.087), and a trend to significance for the interaction between session and time (F_1,15_ = 2.9, p = 0.1).

#### Glx

**Figure 5:**
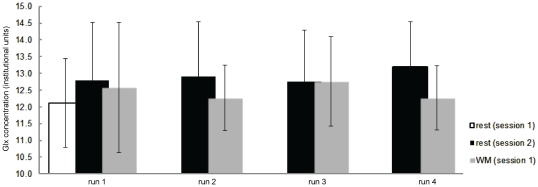
Changes in the Glx (glutamine plus glutamate) neurotransmitter concentration (with standard deviations) during session-specific rest (session 1: open bar, session 2: solid black bar) and working memory (session 1 only: solid grey bar) measurements.

In contrast to GABA, the average Glx concentration did not change significantly between the baseline (session 1) resting and WM1 level (two-tailed t-test, Fig. 5) or with time over subsequent WM runs (one-way rm-ANOVA with factor time). A two-by-four rm-ANOVA with the factors session and time revealed no main effect or interactions for Glx. No significant correlations were evident between Glx or GABA and the grey matter fraction (range across subjects: 25-38%) of the MRS voxel (p = 0.4).

#### Neurotransmitters and perfusion

**Figure 6:**
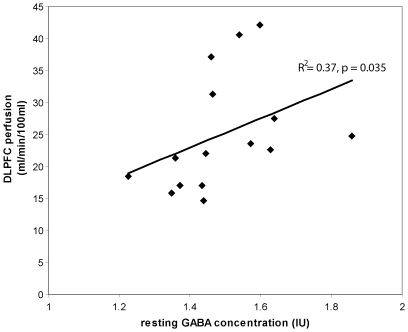
Correlation between resting GABA and resting perfusion in the DLPFC voxel.

The resting GABA level from session 1 correlated positively with the resting DLPFC perfusion (p = 0.035, R^2^ = 0.37, Fig. 6), and inversely with the change in DLPFC perfusion during the WM task (p = 0.032, R^2^ = 0.22). No significant correlation was evident between the resting Glx level and resting or task-related DLPFC perfusion. For session two, there was no significant difference in GABA, glutamate, or Glx between the eyes open and eyes closed resting conditions (p>0.3).

## Discussion

This study demonstrates a local modulation in inhibitory (GABA) neurotransmitter levels between rest and WM, and also reveals continuous changes in GABA during the repetition of the same WM task. These changes in the inhibitory neurotransmitter concentration over time were not seen during repetition of resting MRS measurements.

### Neurotransmitters and WM

There is long-standing evidence that neuronal microcircuits, involving glutamatergic pyramidal neurons and local GABA-ergic interneurons within the prefrontal cortex, have essential roles in the encoding and maintenance of information in WM [55], [56], and prefrontal GABA depletion in the monkey has been shown to induce deficits in delay-task performance [57]. Evidence from neurophysiological studies indicates that (short-term) WM is maintained by persistent cell firing [56], [58], while long-term memory appears to be maintained by modifications in synaptic strength [59]. In our study subjects had to perform a 40 min task, interrupted only by short breaks of less than a minute. Since half of the trial reflects the retention interval and since current models of WM suggest that the DLPFC is predominately active during retention [36], it is likely that the observed changes in the neurotransmitter concentration reflect the cognitive demands of the retention interval. The high level of accuracy demonstrates that our five second retention interval was not too long for the subjects to retain the stimulus set letters actively in memory, which is consistent with results from previous WM studies [60].

The increase in GABA during the first WM task relative to the baseline level could arise either from an increase in GABA release or a decrease in GABA uptake. However, the concurrent increase in Glx, although not significant, suggests that the former is more likely than the latter, as a decrease in GABA uptake would be likely to cause a decrease in Glx levels arising from a decrease in Gln production from the astrocytic GABA-Glu-Gln cycle. Rather, the simultaneous increase in GABA during the first WM task suggests that this modulation of GABA-ergic activity consists of increased GABA release and a general up-regulation of the GABA/Glu/Gln cycle and associated metabolic processes, as hypothesised in earlier studies [61].

Compared to an initial activation period, decreases in metabolite concentration (namely lactate and glutamate) have been shown in the occipital cortex during repeated visual stimulation at 7T [19], [25], [62], [63], [64]. Interestingly, the same study showed no modulation in the BOLD effect with repeated visual stimulation. However, while changes in the activation inhibition balance may alter energy demands (reflected in lactate levels), if the total activity remains constant the magnitude of the BOLD signal may remain unchanged [62]. Alternatively, the difference in the BOLD and lactate response to repeated stimuli may reflect a “metabolic adaptation”, whereby the glycolytic and TCA cycle rates alter but neural firing remains unaffected [62]. Based on the present study it is not possible to decide which of these explanations is more plausible. However, studies incorporating measurements of neuronal activity in addition to MRS and fMRI changes during stimulation may be able to elucidate the physiological basis for these findings.

### The Link between Neurotransmitter Levels and Perfusion

We found increases in perfusion in the DLPFC (and other areas) during the WM task compared to rest (Fig. 3). This observation is consistent with WM-related fMRI studies from our own and other groups, who reported (load-specific) BOLD signal changes in the DLPFC and other regions during WM [65], [66], [67], [68]. In addition, this study provides further evidence for a link between the baseline cerebral blood flow and the baseline GABA concentration (Fig. 6). The positive correlation observed between resting GABA levels and resting perfusion is consistent with results from a recent study reporting a positive association between resting GABA and ASL perfusion in the human visual cortex [13]. In this study we also observed that subjects with high resting perfusion and GABA levels demonstrated a smaller change in perfusion during the WM task, suggesting that subjects with a high baseline neuronal activity require less up-regulation of local hemodynamics during cognition. This coupling is likely to reflect a complex inter-dependence between neurotransmitter and hemodynamic activity, since neurotransmitter cycling processes depend on the citric acid (TCA) cycle, glucose metabolism, and the local availability of oxygen supplied via perfusion, but neurotransmitter release is also thought to represent a key component of the signalling processes involved in vasodilation, and vasoconstriction, and the regulation of perfusion [26].

### The Interaction between Behaviour and Neurotransmitter Concentration

These observed changes in neurotransmitter concentration could potentially be confounded by drowsiness or learning effects. While increased drowsiness would be expected to cause decreased performance and increased reaction times across subjects and task repetitions, habituation or learning effects would be expected to cause the opposite effect. Although we did not observe significant differences in task accuracy or RT between the first and last WM task, we found that RT decreased across WM runs. In addition, we noted a trend towards a positive correlation between RT and GABA, i.e., slower RT’s were paralleled by higher levels of GABA. The inverse correlation between task accuracy and GABA might additionally indicate that a lower level of GABA is linked to higher task accuracy. Therefore, we suggest that the decrease in GABA (Fig. 4) across the four consecutive WM task runs is unlikely to be driven by an increased level of drowsiness, but might reflect learning. We conclude that as individuals become more proficient with the task, task execution becomes more automatic, i.e., less mental effort is needed to perform equally well [69]. In addition, since no comparable decrease in GABA was observed during the four consecutive resting spectra acquired in session 2, we would argue that a change in GABA over time during the task cannot be driven by drowsiness. However, future studies will be needed to clarify whether the level of GABA is further reduced with longer task duration or with higher cognitive loads than those used in this study, both in healthy individuals and individuals with low baseline GABA levels (e.g. disease-related). If drowsiness or task-difficulty effects were to overcome learning effects, the opposite correlations between performance and GABA level would be expected. Nevertheless, despite the fact that the performance is not affected during the task, given the length of the stimulation paradigm neuronal habituation effects cannot be excluded, and may account for the return to baseline of the GABA levels during repetition of the task. The findings should therefore be replicated with a larger number of subjects and under different task conditions.

### Limitations

It is important to note that the GABA levels measured in this study will also contain some contribution from co-edited macromolecule (MM) signals, which can account for nearly half the apparent GABA concentration. However, since the relative contribution of the MM to the GABA+ peak is thought to remain relatively stable [70], we believe the MM signal is unlikely to account for the observed within-session changes in GABA during the WM task. Since perfusion levels are significantly higher in grey matter than in white matter, and since grey matter is also associated with a higher GABA concentration [71] the link between resting perfusion and GABA could also be confounded by variations in the fraction of grey matter across the subject group. However, in our sample, since the grey matter fraction within the MEGA-PRESS voxel was not significantly correlated with the GABA or Glx concentrations, the significant interaction between GABA and perfusion does not appear to be driven by differences in voxel composition across the subject group.

The Glx signal arises mostly from glutamate, which is the primary excitatory neurotransmitter in the brain. However, glutamate is involved in a number of metabolic roles and in addition to functioning as a neurotransmitter it is also an important link between the TCA cycle and amino acid synthesis, a component of the malate-aspartate shuttle, a protein amino acid, and a precursor to GABA [13], [72]. Since the MRS glutamate signal contains contributions from all the glutamate pools, it is not possible to separate the spectral contributions arising from the neurotransmitter population of glutamate from those arising from the other glutamate pools. In addition, since the voxel of interest included a large amount of white matter it is also not possible to state with certainty that the observed changes in neurotransmitter levels arise solely from the grey matter fraction (approximately 30% of the voxel volume) or from the eloquent cortex within the voxel (approximately 10-15% of the voxel volume, estimated from the volume of perfusion change during WM). However future studies incorporating spectral editing with spectroscopic imaging [73], [74] should enable smaller effective voxel sizes, which may help to clarify the specificity of these changes with regard to voxel content.

Although the LCModel basis set included simulated basis spectra for glutathione as well as for GABA, due to the spectral overlap between these metabolites we cannot fully exclude the possibility that glutathione was mis-attributed to GABA (or vice versa). Glutathione was reliably detected for most subjects but we have not looked at changes in glutathione during the task. Future studies will therefore be needed to examine changes in glutathione during cognitive performance.

One could argue that an alternative control strategy, such as using a different voxel location with the same WM task, or using the same voxel location with a different task, might offer some advantages over the resting control session used in this study. However, in a previous study we have shown that the same task elicits activation bilaterally in occipital cortex and parietal cortex during the encoding phase, as well as motor regions during the retrieval period [66]. Therefore, given the large voxel size necessary for adequate signal to noise for the MEGA-PRESS spectra, it is difficult to select a control voxel location which would be completely unaffected by the task. In addition, GABA levels have been shown to vary between brain regions, in a manner which is only partially explained by the local proportion of grey and white matter [75] so different voxel locations would give different baseline neurotransmitter levels. Using a different task with the same voxel location as a control is similarly problematic, since the DLPFC can also be activated by attention tasks, and any control task will necessarily contain some attention component. For these reasons, for the present study we opted to use the same voxel location but repeated resting spectra as a control. However, future studies may be able to clarify whether the change of the neurotransmitter concentration seen here depends on the cognitive load, which would more directly reflect the demands of cognitive/WM processing.

### Conclusions

The initial increase and subsequent reduction of GABA during WM seen in this study suggests a task-dependent local modulation of GABA-ergic activity relative to resting neurotransmitter levels. The correlation between the resting GABA levels and both the resting perfusion and the change in perfusion during WM further suggests a tight coupling between neurotransmitter levels and local hemodynamic changes.
